# Supplementary material for: Natural Products from Singapore Soil-Derived Streptomycetaceae Family and Evaluation of Their Biological Activities
Source: Molecules. 2023 Aug 2;28(15):5832. doi: 10.3390/molecules28155832 (PMC10421265; doi:10.3390/molecules28155832)
Supplement: Supplementary file 1 [file molecules-28-05832-s001.zip › molecules-2502341-supplementary.pdf]

Supplementary information for

Natural Products from Singapore Soil-Derived *Streptomycetaceae* Family and Evaluation of Their Biological Activities

Elaine-Jinfeng Chin †, Kuan-Chieh Ching †, Zann Y. Tan, Mario Wibowo, Chung-Yan Leong, Lay-Kien Yang, Veronica W. P. Ng, Deborah C. S. Seow, Yoganathan Kanagasundaram * and Siew-Bee Ng *

Singapore Institute of Food and Biotechnology Innovation (SIFBI), Agency for Science, Technology and Research (A*STAR), Singapore 138673, Singapore; elainec@sifbi.a-star.edu.sg (E.-J.C.); chingkc@sifbi.a-star.edu.sg (K.-C.C.); tanyq@sifbi.a-star.edu.sg (Z.Y.T.); mario_wibowo@sifbi.a-star.edu.sg (M.W.); leongcy@sifbi.a-star.edu.sg (C.-Y.L.); yanglk@sifbi.a-star.edu.sg (L.-K.Y.); ngwp@sifbi.a-star.edu.sg (V.W.P.N.); seowcs@sifbi.a-star.edu.sg (D.C.S.S.)

* Correspondence: yoganathank@sifbi.a-star.edu.sg (Y.K.); ngsb@sifbi.a-star.edu.sg (S.-B.N.); Tel.: +65-62792716 (Y.K.); +65-62792727 (S.-B.N.)

† These authors contributed equally to this work.

Figure S1. UV spectrum for **1**.

Figure S2. (+)-HRESIMS spectrum for **1**.

Figure S3. ^{1}H NMR spectrum ($(CD_3)_2CO$, 400 MHz) of **1**.

Figure S4. COSY spectrum of **1**.

Figure S5. HSQC spectrum of **1**.

Figure S6. HMBC spectrum of **1**.

Figure S7. ^{1}H NMR spectrum ($(CD_3)_2CO$, 400 MHz) of tetronomycin.

Figure S8. HSQC spectrum of tetronomycin.

Figure S9. HMBC spectrum of tetronomycin.

Figure S10. Dose response curve against *Staphylococcus aureus* Rosenbach (SA25923), *Klebsiella aerogenes* (EA13048), *Pseudomonas aeruginosa* (PA9027), *Candida albicans* (CA10231) and *Aspergillus fumigatus* (AF46645). A) Nonactin, B) Monactin, C) Dinactin, D) 4E-Deacetylchromomycin A3, E) Chromomycin A2, F) Lysolipin I, G) Soyasaponin II and H) Naphthomevalin.

Figure S11. Dose response curve against A549 human lung carcinoma cells, and two pancreatic cancer cell lines MIA PaCa-2 and PANC-1 cells. A) Nonactin, B) Monactin, C) Dinactin, D) 4E-Deacetylchromomycin A3, E) Chromomycin A2, F) Lysolipin I, G) Soyasaponin II and H) Naphthomevalin.

Figure S12. Dose response curve against *Klebsiella aerogenes* (EA13048), *Pseudomonas aeruginosa* (PA9027), *Candida albicans* (CA10231) and *Aspergillus fumigatus* (AF46645). A) **1** and B) Tetronomycin.

Table S1. Antimicrobial and cytotoxicity primary screening results of 4 actinobacteria strains grown in 5 different growth media.

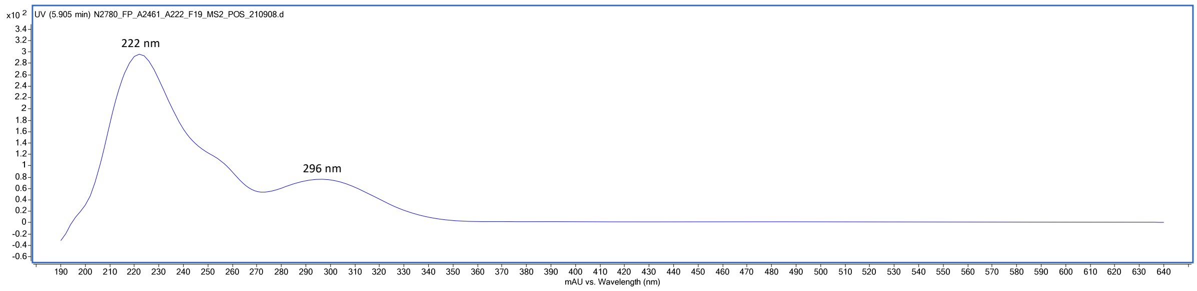

Figure S1. UV spectrum for **1**.

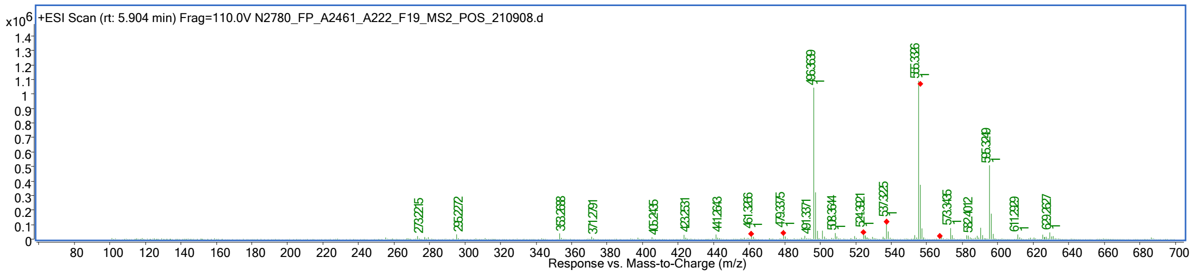

Figure S2. (+)-HRESIMS spectrum for **1**.

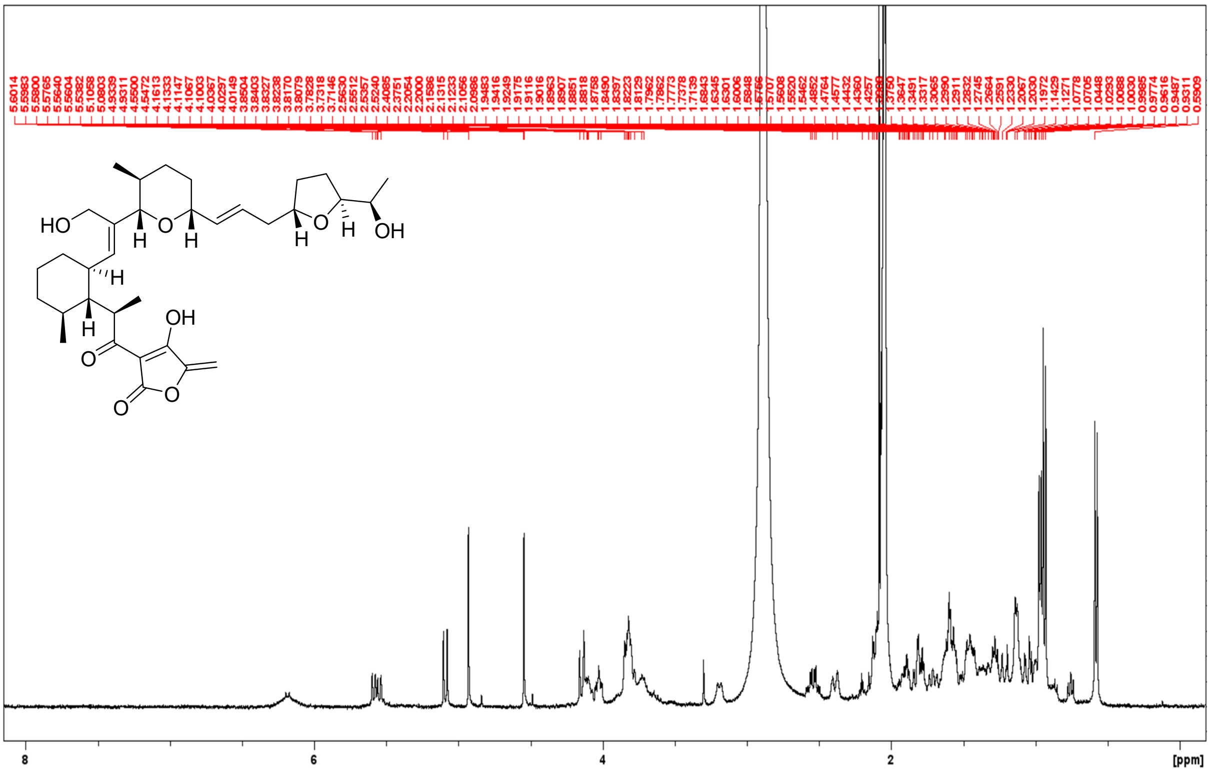

Figure S3. ^{1}H NMR spectrum ($(CD_3)_2CO$, 400 MHz) of **1**.

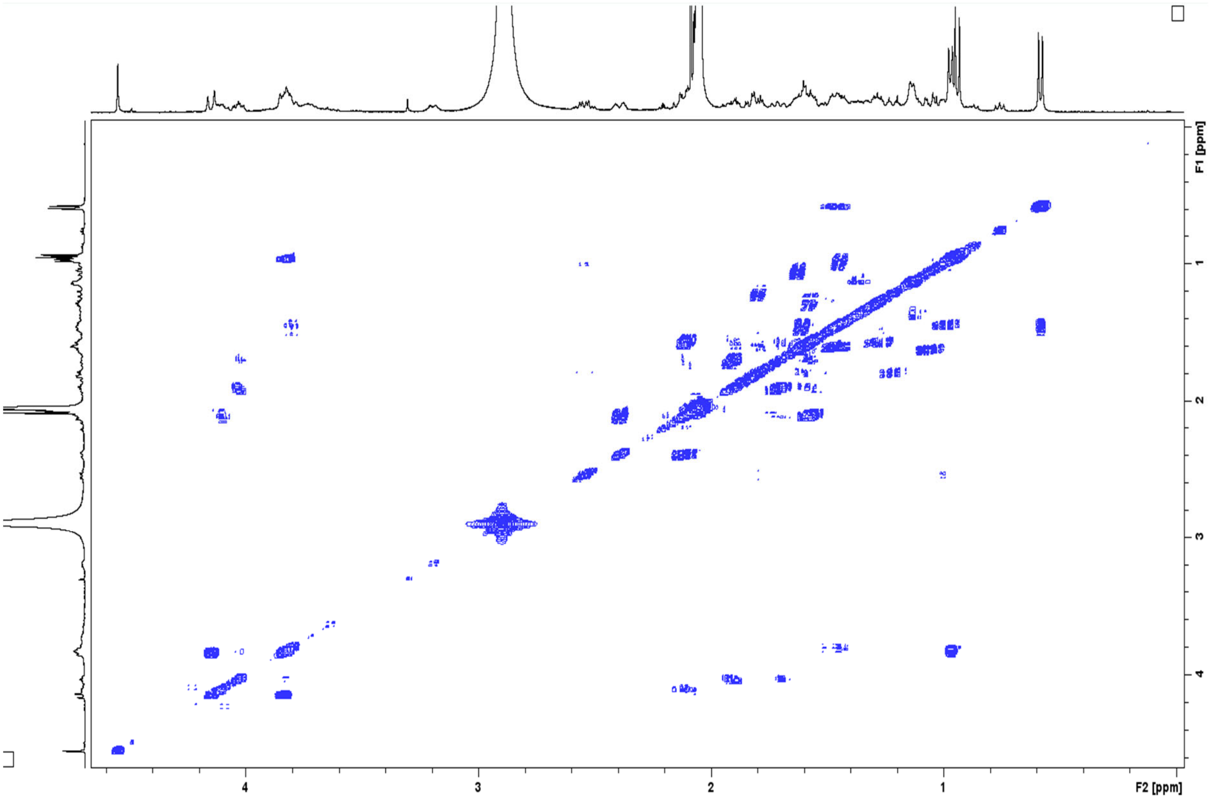

Figure S4. COSY spectrum of **1**.

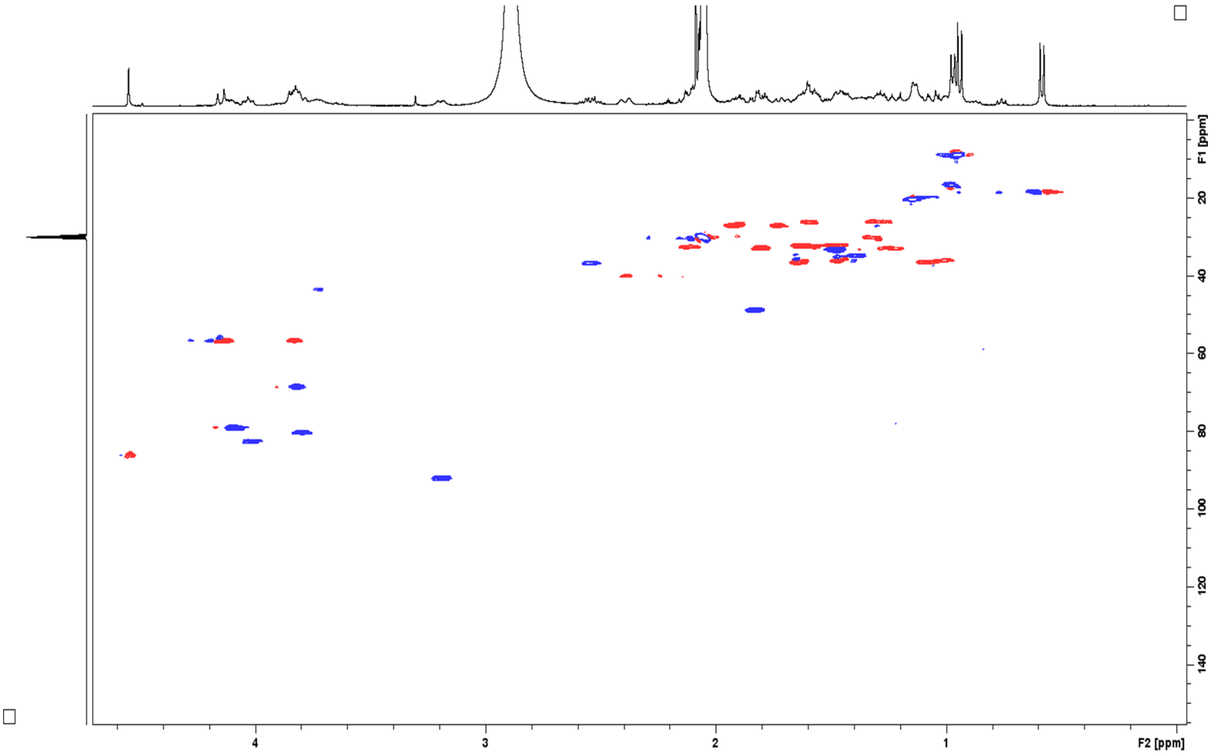

Figure S5. HSQC spectrum of **1**.

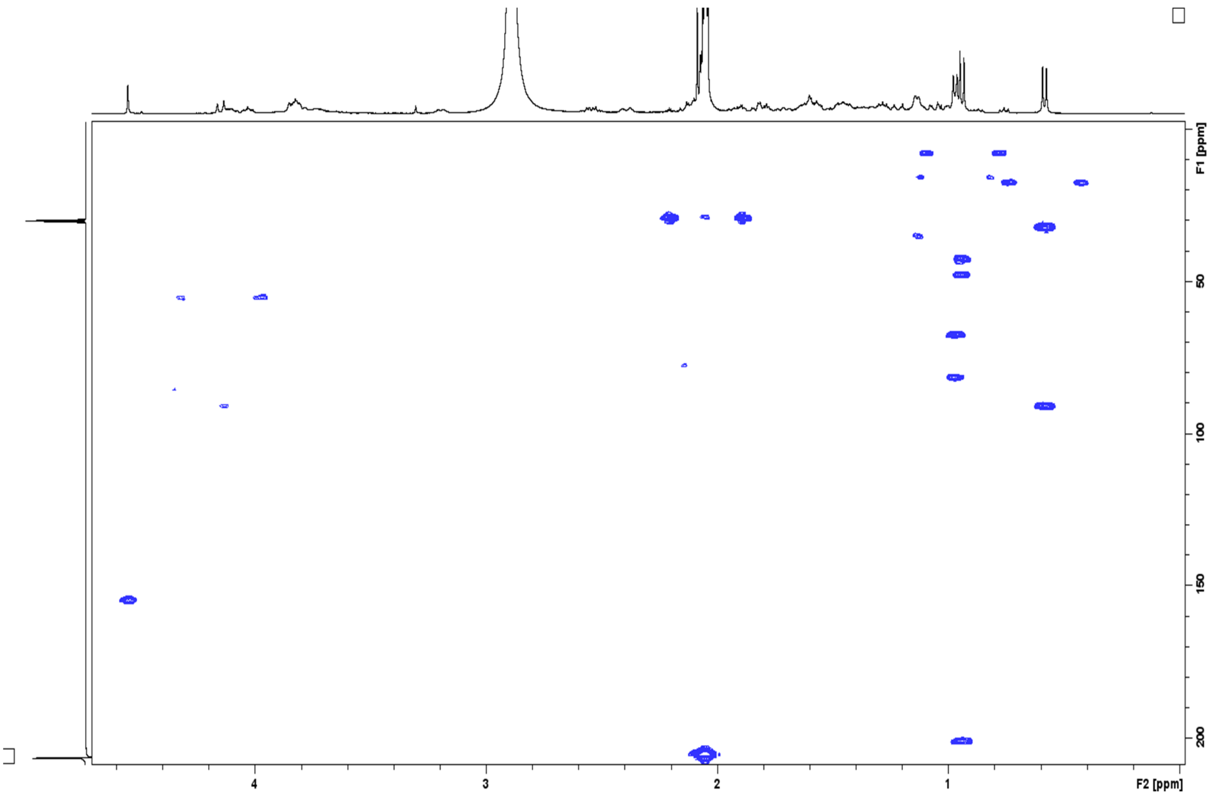

Figure S6. HMBC spectrum of **1**.

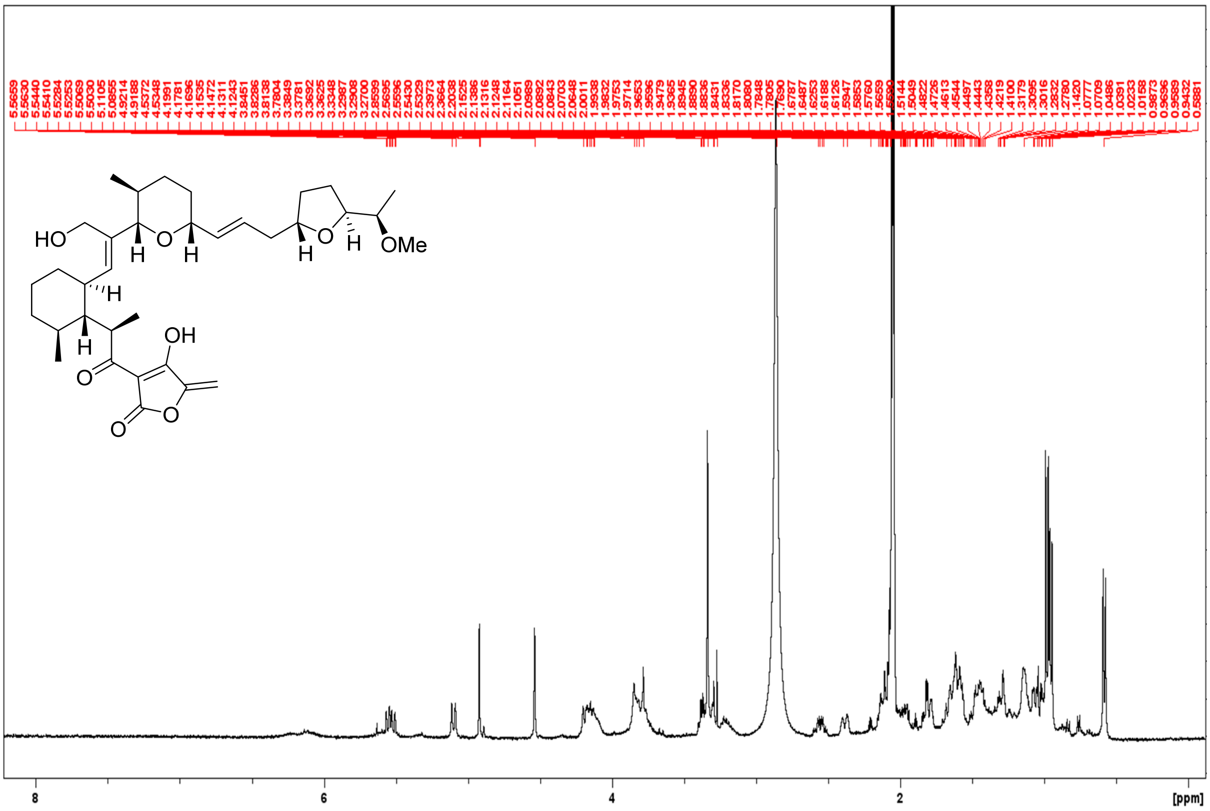

Figure S7. ^{1}H NMR spectrum ($(CD_3)_2CO$, 400 MHz) of tetronomycin.

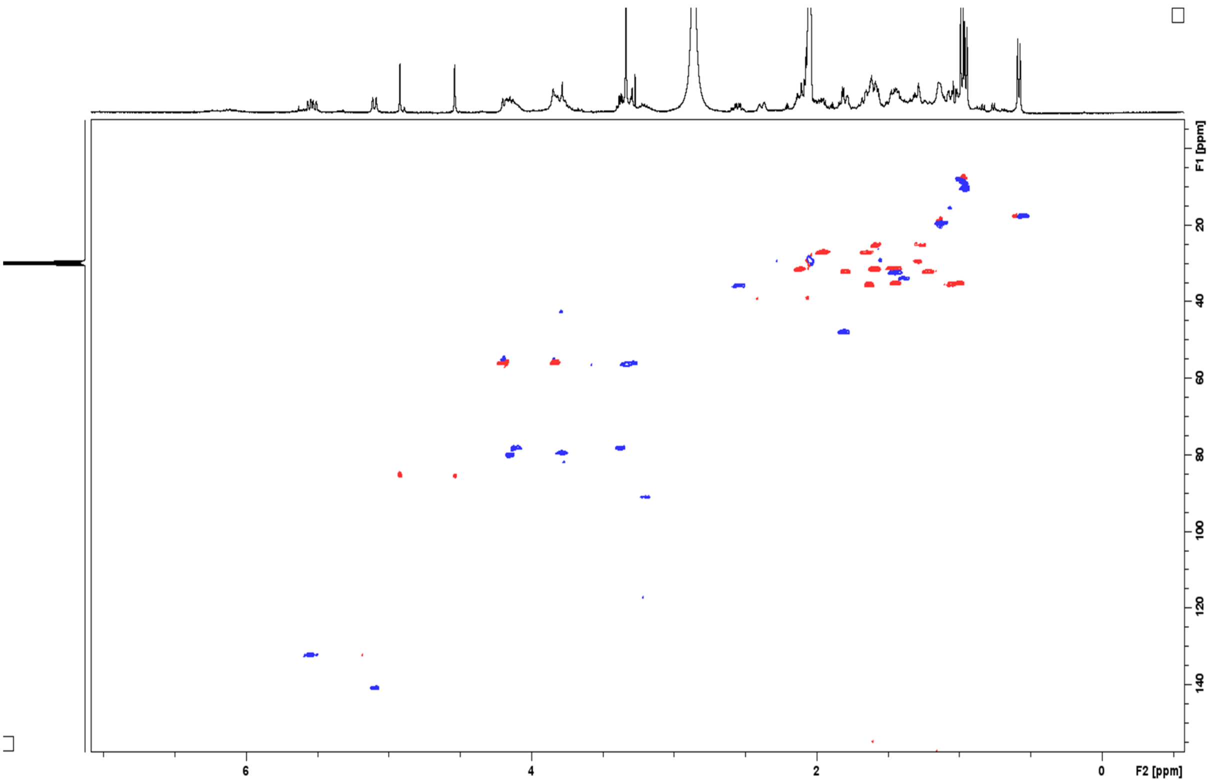

Figure S8. HSQC spectrum of tetronomycin.

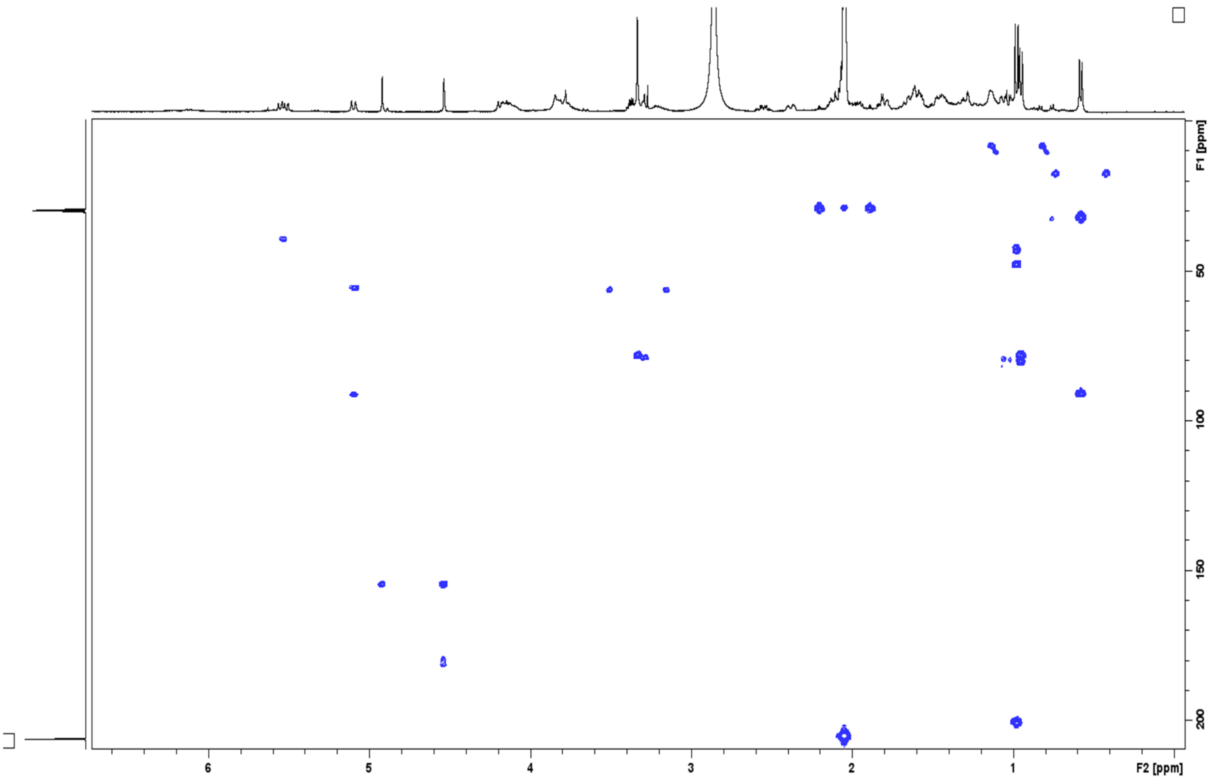

Figure S9. HMBC spectrum of tetronomycin.

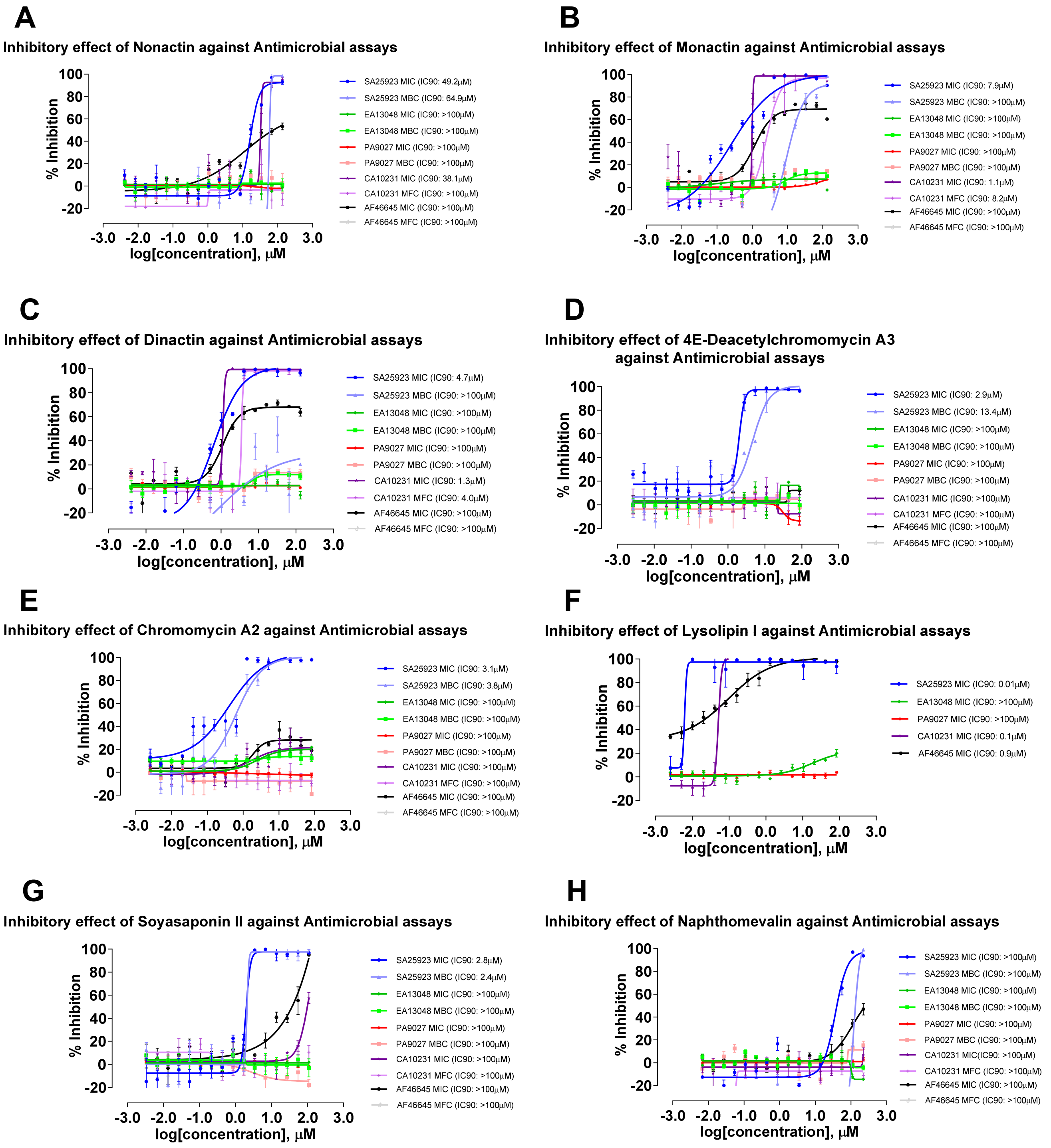

Figure S10. Dose response curve against *Staphylococcus aureus* Rosenbach (SA25923), *Klebsiella aerogenes* (EA13048), *Pseudomonas aeruginosa* (PA9027), *Candida albicans* (CA10231) and *Aspergillus fumigatus* (AF46645). A) Nonactin, B) Monactin, C) Dinactin, D) 4E-Deacetylchromomycin A3, E) Chromomycin A2, F) Lysolipin I, G) Soyasaponin II and H) Naphthomevalin.

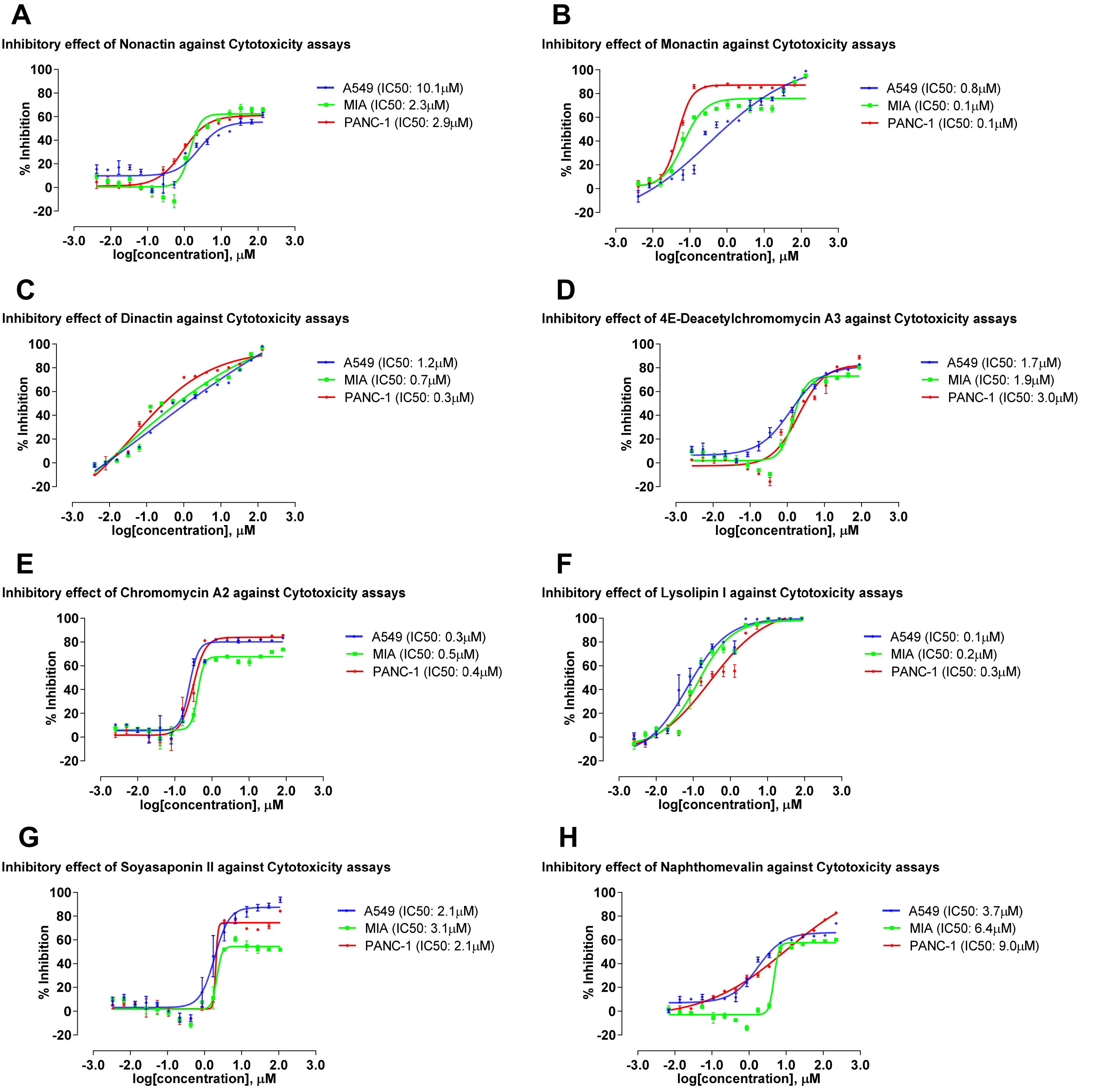

Figure S11. Dose response curve against A549 human lung carcinoma cells, and two pancreatic cancer cell lines MIA PaCa-2 and PANC-1 cells. A) Nonactin, B) Monactin, C) Dinactin, D) 4E-Deacetylchromomycin A3, E) Chromomycin A2, F) Lysolipin I, G) Soyasaponin II and H) Naphthomevalin.

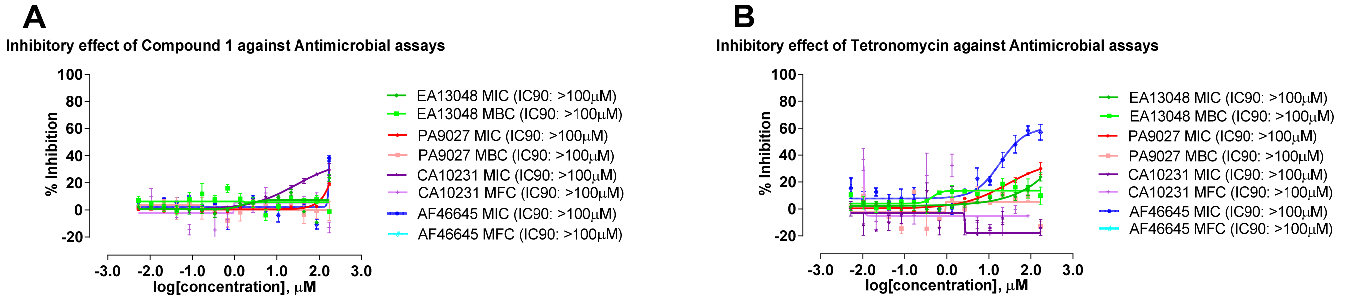

Figure S12. Dose response curve against *Klebsiella aerogenes* (EA13048), *Pseudomonas aeruginosa* (PA9027), *Candida albicans* (CA10231) and *Aspergillus fumigatus* (AF46645). A) **1** and B) Tetronomycin.

Table S1. Antimicrobial and cytotoxicity primary screening results of 4 actinobacteria strains grown in 5 different growth media.

Samples	Media	Antimicrobial (% Inhibition)					Cytotoxicity (% Inhibition)		
		KA	PA	SA	CA	AF	A549	MIA	PANC-1
A1099	CA02LB	<80	<80	85.9	96.7	<80	<80	<80	<80
	CA07LB	<80	<80	88.9	96.3	<80	<80	<80	<80
	CA08LB	<80	<80	99.0	99.7	<80	<80	<80	<80
	CA09LB	<80	<80	<80	<80	<80	<80	<80	<80
	CA10LB	<80	<80	<80	<80	<80	<80	<80	<80
A1174	CA02LB	<80	<80	<80	<80	<80	<80	<80	<80
	CA07LB	<80	<80	<80	<80	<80	<80	<80	<80
	CA08LB	<80	<80	98.1	<80	<80	81.8	<80	<80
	CA09LB	<80	<80	<80	<80	<80	<80	<80	<80
	CA10LB	<80	<80	<80	<80	<80	<80	<80	<80
A1301	CA02LB	<80	<80	<80	<80	97.0	<80	<80	<80
	CA07LB	<80	<80	80.9	92.8	<80	99.4	98.8	96.3
	CA08LB	<80	<80	99.5	<80	<80	<80	<80	<80
	CA09LB	<80	<80	99.5	<80	<80	<80	<80	<80
	CA10LB	<80	<80	92.2	98.9	95.5	98.9	96.3	95.6
A2461	CA02LB	<80	<80	<80	<80	<80	<80	<80	<80
	CA07LB	<80	<80	<80	<80	<80	<80	<80	<80
	CA08LB	<80	<80	98.5	<80	<80	<80	<80	<80
	CA09LB	<80	<80	<80	<80	<80	<80	<80	<80
	CA10LB	<80	<80	99.1	<80	<80	<80	<80	<80

[1]KA = *Klebsiella aerogenes*, PA = *Pseudomonas aeruginosa*, SA = *Staphylococcus aureus* Rosenbach, CA = *Candida albicans* and AF = *Aspergillus fumigatus*.
[2]A549 = human lung carcinoma cells, MIA = pancreatic cancer cells and PANC-1 = pancreatic cancer cells.
Antimicrobial effect and cytotoxic activity (average growth inhibition ≥ 80%).
